# DynamicSleepNet: a multi-exit neural network with adaptive inference time for sleep stage classification

**DOI:** 10.3389/fphys.2023.1171467

**Published:** 2023-05-10

**Authors:** Wang Wenjian, Xiao Qian, Xue Jun, Hu Zhikun

**Affiliations:** School of Information Science, Yunnan University, Kunming, China

**Keywords:** multimodal, sleep stage, depth-adaptive, feature fusion, attention network, electrophysiological signals

## Abstract

Sleep is an essential human physiological behavior, and the quality of sleep directly affects a person’s physical and mental state. In clinical medicine, sleep stage is an important basis for doctors to diagnose and treat sleep disorders. The traditional method of classifying sleep stages requires sleep experts to classify them manually, and the whole process is time-consuming and laborious. In recent years, with the help of deep learning, automatic sleep stage classification has made great progress, especially networks using multi-modal electrophysiological signals, which have greatly improved in terms of accuracy. However, we found that the existing multimodal networks have a large number of redundant calculations in the process of using multiple electrophysiological signals, and the networks become heavier due to the use of multiple signals, and difficult to be used in small devices. To solve these two problems, this paper proposes DynamicSleepNet, a network that can maximize the use of multiple electrophysiological signals and can dynamically adjust between accuracy and efficiency. DynamicSleepNet consists of three effective feature extraction modules (EFEMs) and three classifier modules, each EFEM is connected to a classifier. Each EFEM is able to extract signal features while making the effective features more prominent and the invalid features are suppressed. The samples processed by the EFEM are given to the corresponding classifier for classification, and if the classifier considers the uncertainty of the sample to be below the threshold we set, the sample can be output early without going through the whole network. We validated our model on four datasets. The results show that the highest accuracy of our model outperforms all baselines. With accuracy close to baselines, our model is faster than the baselines by a factor of several to several tens, and the number of parameters of the model is lower or close. The implementation code is available at: https://github.com/Quinella7291/A-Multi-exit-Neural-Network-with-Adaptive-Inference-Time-for-Sleep-Stage-Classification/.

## 1 Introduction

Sleep is an essential physiological behavior for human beings, and the quality of sleep directly affects a person’s physical and mental state. Studies show that people who have quality sleep also enjoy a healthy body and an energetic mind (Luyster et al., 2012). However, as the pace of life accelerates and the stress of life increases, many people suffer from sleep problems, such as sleep interruption and insomnia (Cesari et al., 2021). In sleep medicine, doctors can measure the quality of sleep by monitoring the patient’s sleep stages for better treatment of patients with sleep problems (Pan et al., 2020). But until today, it is still a challenge for doctors to efficiently classify the sleep stages.

Polysomnography (PSG) is capable of collecting many physiological parameters during sleep and is considered by sleep experts to be the gold standard for measuring sleep quality and sleep disorders (Rundo and Downey, 2019; Malekzadeh et al., 2022). In general, PSG collects electroencephalography (EEG), electrooculography (EOG), electromyography (EMG) and electrocardiography (ECG) and other electrophysiological signals (Pandi-Perumal et al., 2014), and then PSG is divided into separate 30s epochs. According to the American Academy of Sleep Medicine (AASM) standard, epochs can be categorized into five different sleep stages: wake (W); Non-REM1 (N1); Non-REM2 (N2); Non-REM3 (N3); and rapid eye movement (REM) (Berry et al., 2012). The traditional classification method requires manual division by sleep experts, which not only requires the use of a lot of expertise but is also very time-consuming and highly subjective. Therefore, it is imperative to study methods for automatic sleep stage classification.

Traditional machine learning algorithms are first applied to automatic sleep classification, and these methods usually need manual feature extraction, such as support vector machines (SVM) (Zhu et al., 2014; Seifpour et al., 2018), Naive Bayes (Dimitriadis et al., 2018), random forests (RF) (Li et al., 2017), and ensemble learning based classifiers (Hassan and Bhuiyan, 2016). Although these models have achieved some results, the feature extraction requires some prior expertise, resulting in poor transfer ability of these models (Eldele et al., 2021). In recent years, deep learning has gradually gained popularity among researchers due to the excellent performance it has shown and the low need for prior expertise. Many researchers have proposed effective deep learning models for automatic sleep classification. Some researchers have used convolutional neural networks (CNNs) for feature extraction of EEG, EOG, and EMG signals to achieve sleep stage classification (Phan et al., 2018b; Yildirim et al., 2019; Zhang et al., 2020), some researchers have used the ability of recurrent neural networks (RNNs) to extract features of both forward and reverse sequences for classification (Phan et al., 2018a; Phan et al., 2019; Sun et al., 2019), some researchers have used the property of U-Net networks to handle cyclic alternating patterns (CAP) for classification, and some researchers have taken advantage of the Transformer’s ability to model the relationship between global inputs and outputs for classification (Eldele et al., 2021; Phyo et al., 2022; Zheng et al., 2022) The researchers tried to propose models with higher accuracy for sleep stage classification, and some problems have arisen as a result.

**FIGURE 1:**
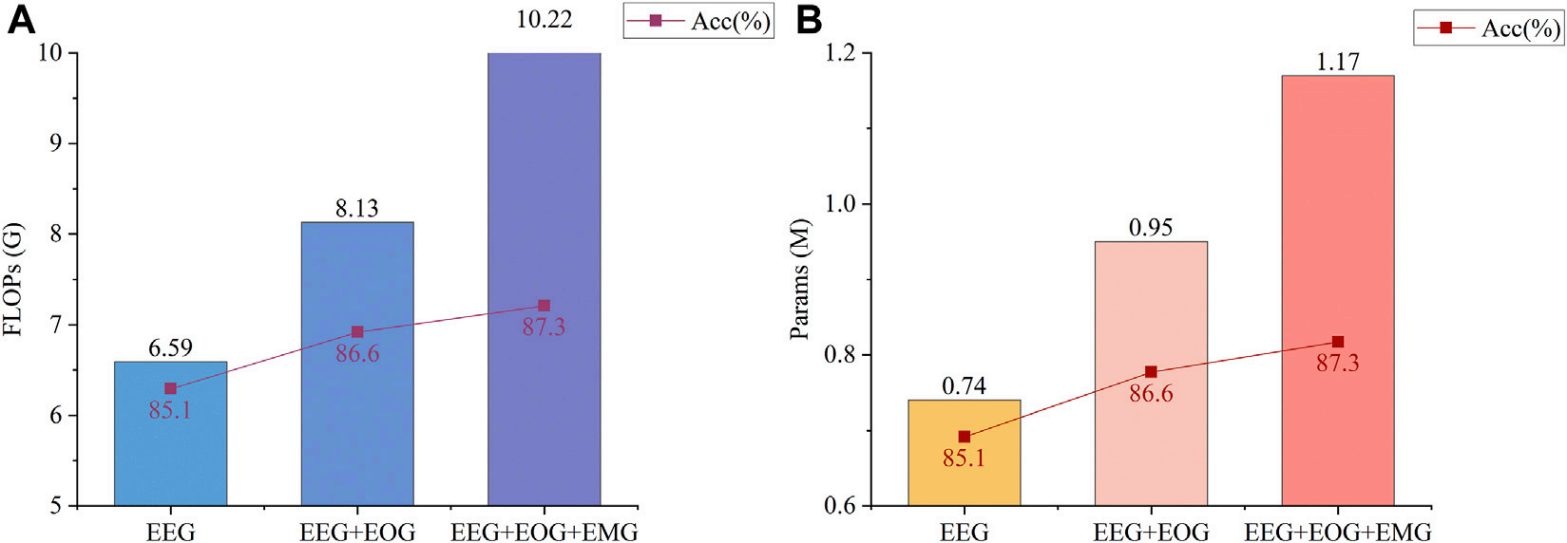
Metrics of MMASleepNet (Zheng et al., 2022) runs on the Sleep-EDF-20 dataset. The accuracy data for the three conditions are from Section 4.2 of the original paper, and the FLOPs and Params are obtained by running its open source code. The source code of the model has not been changed. **(A)** shows the FLOPs and Accuracy of the model for three different input conditions (EEG, EEG + EOG, and EEG + EOG + EMG). The batch size of the input is 256. **(B)** shows the Params and Accuracy of the model for three different input conditions (EEG, EEG + EOG, EEG + EOG + EMG). The batch size of the input is 256.

In some recent studies, multimodal networks are very popular among researchers (Jia et al., 2021; Pradeepkumar et al., 2022; Zheng et al., 2022) Multimodal networks can accept different kinds of signals as input (e.g., EEG, EOG, EMG) and assign weights according to the importance of different signals to the current sample classification, which improves the classification accuracy compared to unimodal networks that use only one kind of signal as the classification basis. Despite the improvement in accuracy of these models, the greater computational cost in order to compute multimodal signals is often overlooked, which means that these models are difficult to use in practice. As an example, MMASleepNet (Zheng et al., 2022) is a multimodal model that has recently been designed for sleep stage classification. The accuracy improvement from using multimodal signals in MMASleepNet *versus* the increased floating-point operations (FLOPs) and params is shown in Figure 1. In fact, it can be seen from Figure 1 that the accuracy improvement with the addition of EOG and EMG signals is limited. The accuracy data are from Section 4.2 of the MMASleepNet paper, and the FLOPs and Params are calculated by us using the open source code of the original paper. The source code of the model has not been changed. In other words, many samples are sufficient for classification using EEG signals alone, while only those samples that are difficult to classify using EEG signals alone need to be classified with the help of EOG and EMG signals. Most multimodal models may not have considered this.

One approach to improving accuracy, often used in some research works, is to feed only a fixed number of samples (usually dozens of samples) into the model. This allows the model to learn the differences between the samples and thus improve the classification results (Jia et al., 2021; Phan et al., 2021; Pradeepkumar et al., 2022). But this approach presents a challenge for the performance of edge devices. For example, if 20 samples are fed to the edge device at one time, each for 30 s, with a sampling frequency of 100 Hz. In a short period of time, the edge device needs to compute up to 60,000 pieces of data, and this number will be multiplied several times if it is a multimodal network, and devices with low performance are likely to face the problem of running out of memory or computational resources and causing the software to terminate prematurely. Devices that meet the performance requirements need to continuously collect data for 600 s before the next computation, during which no data is available for calculation, and wasting the computational resources of the device.

Each class of samples has its own unique features. CNN is no doubt a powerful feature extraction tool, which can extract different features in each part of the sample. CNN also treats the features of each channel equally, but not every feature extracted is helpful for the classification task, and even the local information of each feature extracted is not equally important for the classification task. Most works simply use CNN as a tool for feature extraction (Sridhar et al., 2020; Supratak and Guo, 2020; Phan et al., 2021; Pradeepkumar et al., 2022) and do not do not take into account the importance of different features.

**FIGURE 2:**
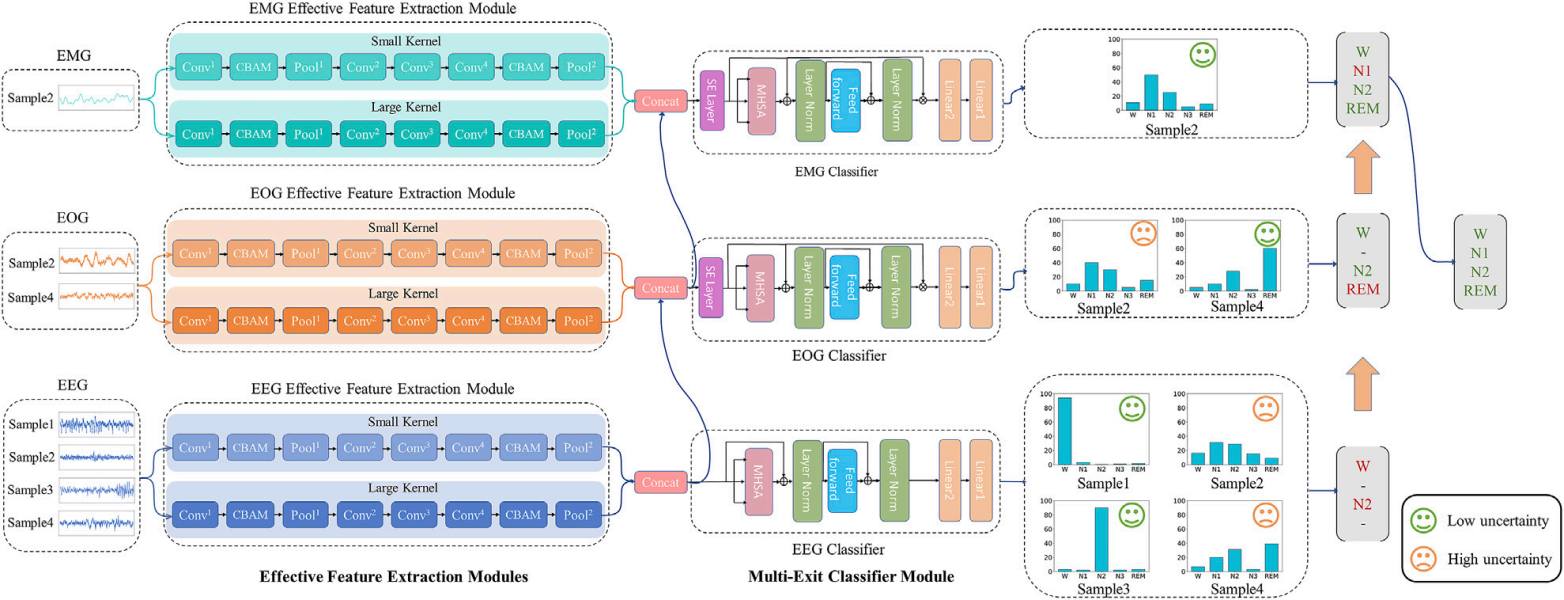
The architecture of our proposed model. The model consists of three effective feature extraction modules (EFEM) and multi-exit classifier modules (MECM). ⊕ is the point-wise addition and ⊗ is the point-wise multiplication. Conv is the convolutional layer, Pool is the pooling layer, MHSA is the multi-head self-attention layer.

To efficiently utilize multimodal signals, reduce unnecessary computations, focus on important features and suppress unnecessary features, we propose the DynamicSleepNet, which consists of three effective feature extraction modules (EFEM) and multi-exit classifier modules (MECM), as shown in Figure 2. The main contributions of this paper can be summarized as follows:

(1) This paper proposes a practical speed-tunable sleep stage classification model, namely DynamicSleepNet, that can be dynamically tuned between computational speed and accuracy to match different performance devices. To the best of our knowledge, this type of speed-tunable dynamic model is proposed for the first time in the field of sleep stage classification.
(2) A sample-wise adaptive mechanism for sleep stage classification is proposed to solve the problem of redundant training of samples for multimodal sleep classification networks. The experimental results on the four public datasets show that DynamicSleepNet has a huge reduction in computational cost with very little loss of accuracy.
(3) This paper develops two auxiliary modules: CBAM block and SE block, which are used to highlight effective features to solve the problem of CNN treating all features equally. The results show that our model outperforms all the baseline models in terms of accuracy, macro F1-Score, and Cohen’s Kappa.

## 2 Materials and methods

### 2.1 Dataset description

In this section, we describe four public datasets Sleep-EDF-78, Sleep-EDF-20, ISRUC-1, and ISRUC-3, used to evaluate the validity of our model. The details of the datasets we used are shown in Table 1. The data we used in the experiments are all original data from the dataset and have not been pre-processed.

**TABLE 1:** The details of the dataset.

Dataset	Subjects	Channel number	Samples	W	N1	N2	N3	REM
Sleep-EDF-78	78	4	195479	65951 (33.7%)	21522 (11.0%)	69132 (35.4%)	13039 (6.7%)	25835 (13.2%)
Sleep-EDF-20	20	4	42308	8183 (19.3%)	2909 (6.9%)	17509 (41.4%)	6006 (14.2%)	7701 (18.2%)
ISRUC-1	100	11	87187	18237 (20.9%)	11110 (12.7%)	27232 (31.2%)	18188 (20.9%)	12420 (14.2%)
ISRUC-3	10	11	8589	1647 (19.2%)	1188 (13.8%)	2660 (31.0%)	1939 (22.6%)	1155 (13.4%)

The Sleep-EDF-78 dataset contains 153 sleep cassette files collected from 78 subjects aged 20–101. Each sleep cassette file includes one whole night of PSG recordings, each recording includes two EEG channels (Fpz-Cz and Pz-Oz), a horizontal EOG channel, and a submental chin EMG channel. The sampling rate is 100 Hz for the EEG and EOG channels, and 1 Hz for the EMG channel. Sleep experts divided the PSG recordings into 30s long epochs according to the Rechtschaffen and Kales (R&K) (Rechtschaffen, 1968) guidelines and manually labeled each epoch as one of the following categories: WAKE, REM, N1, N2, N3, N4, MOVEMENT, UNKNOWN. To be consistent with previous studies (Eldele et al., 2021; Phyo et al., 2022; Pradeepkumar et al., 2022; Zheng et al., 2022), we converted the R&K guidelines to AASM guidelines by combining classes N3 and N4 into a single class N3 and excluding the " MOVEMENT ″ and " UNKNOWN ″ classes. In addition, our study excluded continuous wake epochs longer than 30 min outside of sleep periods, which is also consistent with previous studies. Altogether 195,479 30s epochs of PSG recordings were extracted. Similar to Sleep-EDF-78, the Sleep-EDF-20 dataset collected a total of 42,308 30s epochs from 20 subjects aged 25–34 years.

ISRUC-1 dataset (Khalighi et al., 2016) collected PSG records from 100 subjects aged 20–85 years, each record included six EEG channels, F3-A2, C3-A2, O1-A2, F4-A1, C4-A1, O2-A1, two EOG channels, LOC-A2, ROC-A1, three EMG channels, Chin EMG, left leg movement, and right leg movement. All channels are sampled at 200 Hz and include a total of 87,187 such PSG recordings, each with a length of 30s. The sleep stage to which each record belonged was judged by two sleep experts, and records in which the two experts judged inconsistent are not included in the data of the experiments in this paper. ISRUC-3 collected 8,589 PSG records from 10 healthy subjects aged 30–58 years, consistent with the structure of ISRUC-1. We also excluded records that are judged inconsistent by two experts. For all four datasets above, we used 10-fold cross-validation to obtain our experimental results.

### 2.2 Method

Figure 2 shows the general framework and inference process of DynamicSleepNet. DynamicSleepNet uses a three-step approach, namely “train twice, infer once”. First, only three effective feature extraction (EFEM) modules and EMG Classifier are trained, and all samples are classified only by EMG Classifier. Then we freeze the modules except the EEG Classifier and the EOG Classifier, and train these two classifiers in combination with the self-distillation mechanism. Each sample needs to be trained by each of these three classifiers, but since we freeze most of the model, the actual time spent is much less than the first time. Finally, in the inference stage, for each classifier, we measure for each sample on whether the current inference is credible enough to be terminated. In the following subsections, we will describe in detail the structure of our model and how it works.

#### 2.2.1 Effective feature extraction module

**FIGURE 3:**
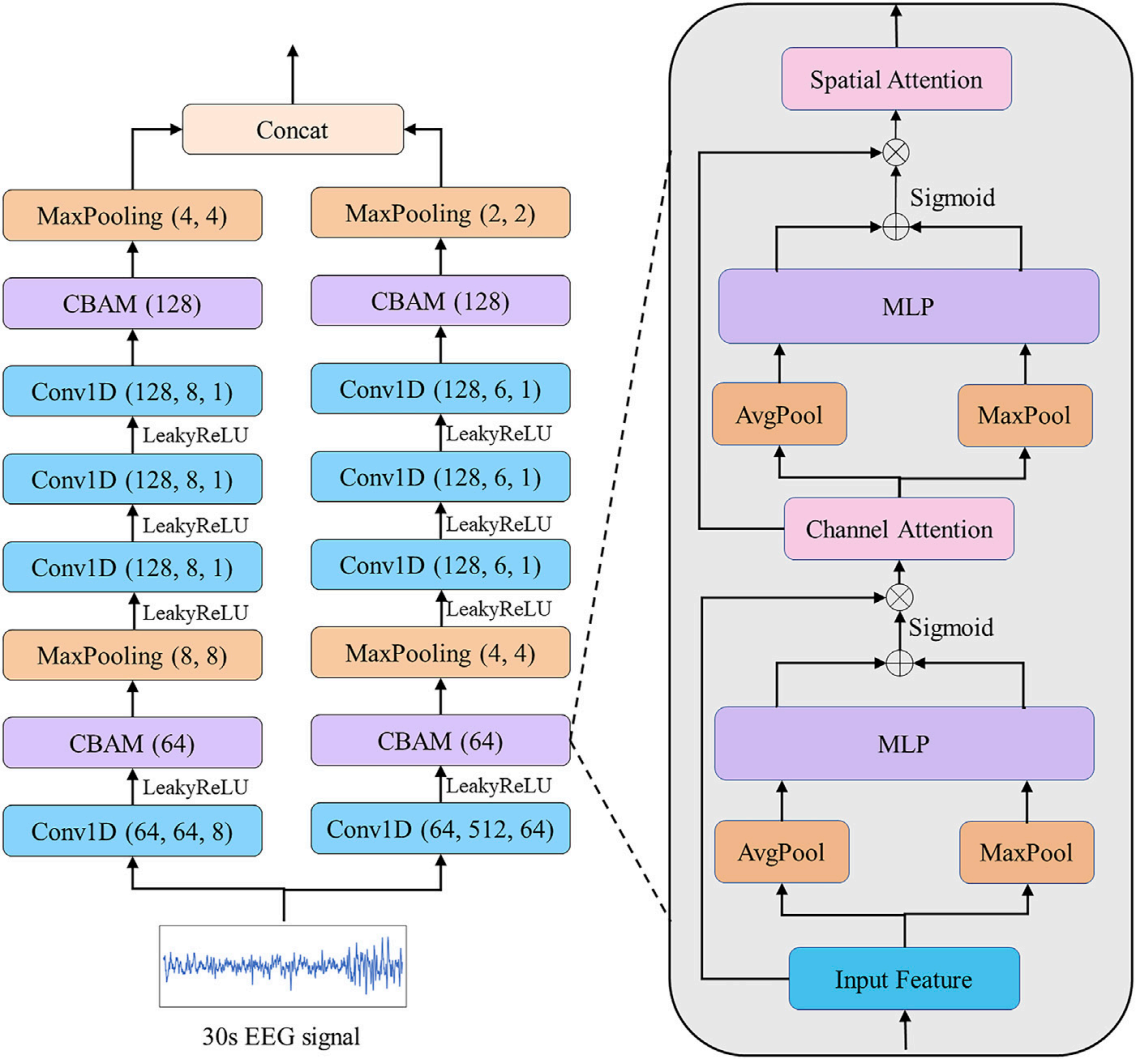
EEG EFEM for extracting effective features. Each convolution block is followed by a Batch Normalization. $Conv1D\;\left(64,64,8\right)$ refers to using 1D convolution layer with 64 filters, a kernel size of 64 and a stride of 8. Similarly, $MaxPooling\;\left(8,8\right)$ refers to a maxpooling layer with a kernel size of 8 and a stride of 8. The leaky rectified linear unit ( Leaky−ReLU ) refers to the activation function of each convolutional layer.

EEG signal is the main method to discriminate different sleep stages. θ wave (4–8 Hz) is stable in the relaxed state with eyes closed and is an effective feature to distinguish Wake(W) stage. α wave (8–13 Hz) is common in the late Non-REM1 (N1) stage and is also a valid feature to distinguish N1 stage. We designed a small convolution kernel with a kernel size of 64, which can capture 0.64s $\left(F=1/T\right)$ of information for each convolution window at a sampling rate of 100Hz, which means we can capture relatively high frequency information like α ; θ waves completely; δ wave (0.5–4 Hz) is the main waveform in Non-REM3 (N3) stage, which is relatively low-frequency information. We designed a large convolution kernel with a kernel size of 512 and each convolution window can capture 5.12s of information, which means we can completely extract the low frequency information like δ wave. The effectiveness of this method of extracting different local features using convolutional kernels of different sizes has been demonstrated in the previous studies (Supratak et al., 2017; Eldele et al., 2021; Pradeepkumar et al., 2022). EOG EFEM, EMG EFEM and EEG EFEM have the same model structure. Taking EEG EFEM as an example, the structure is shown in Figure 3. The hyperparameters of the three EFEMs have been uploaded as supplementary material because the table is too large.

We propose a CBAM block based on CBAM (Woo et al., 2018). The previous CBAM was specifically designed for extracting image features by using 2D convolution and pooling layers. Different from their study, we use 1D convolution and pooling layers for extracting electrophysiological signal features. The CBAM block consists of a channel attention module, which aims to adaptively select the most helpful features for classification, and a spatial attention module, which aims to enhance the most important parts of each feature. Taking the CBAM block described in Figure 3 as an example, given an input $I\in R^{L\times d}$ , we apply a convolution operation to I such that $F=Conv1D\left(I\right)$ , where $F=\left\{F1,\ldots ,F_{C}\right\}\in R^{C\times d}$ , F is the input feature of the CBAM block, C is the total number of features, d is the length of $F_{i}\left(1\leq i\leq C\right)$ , and Conv1D is the convolution operation in EFEM module.

Next, we move to the channel attention module. The global spatial information is squeezed by using global average pooling and global maximum pooling that shrinks $F\in R^{C\times d}$ to $F_{avg}^{c}\in R^{C\times 1}$ and $F_{\max}^{c}\in R^{C\times 1}$ . These two features are fed into a shared multilayer perceptron (MLP) network, which then uses sigmoid as the activation function to produce the channel attention feature map $M_{c}$ . The process is shown in Eq. 1.

$$\begin{array}{c} M_{c}\left(F\right)=\sigma \left(MLP\left(AvgPool\left(F\right)\right)+MLP\left(MaxPool\left(F\right)\right)\right)\\ =\sigma \left(\omega_{1}\left(\omega_{0}\left(F_{avg}^{c}\right)\right)+\omega_{1}\left(\omega_{0}\left(F_{\max}^{c}\right)\right)\right)\in R^{C\times 1} \end{array}$$ (1)

σ refers to the sigmoid activation function, $\omega_{0}$ and $\omega_{1}$ refer to the pooling layer and the MLP layer respectively. Then, the feature map F is scaled by $M_{c}$ as follows:

$$F^{\prime }=M_{c}\left(F\right)⊗F\in R^{C\times d}$$ (2)

where ⊗ is the point-wise multiplication. The feature F is processed by the channel attention module to obtain $F^{\prime }$ , which is the same dimension as F . Next, move to the spatial attention module. The channel information is squeezed by using global average pooling and global maximum pooling that shrinks $F^{\prime }\in R^{C\times d}$ to $F_{avg}^{s}\in R^{1\times d}$ and $F_{\max}^{s}\in R^{1\times d}$ . We concatenate these two features and apply a convolution operation to the concatenated feature, then uses sigmoid as the activation function to produce the spatial attention feature map $M_{s}$ . The process is shown in Eq. 3.

$$\begin{array}{c} M_{s}\left(F^{\prime }\right)=\sigma \left(f^{7}\left(\left[AvgPool\left(F^{\prime }\right);MaxPool\left(F^{\prime }\right)\right]\right)\right)\\ =\sigma \left(f^{7}\left(F_{avg}^{s};F_{\max}^{s}\right)\right)\in R^{1\times d} \end{array}$$ (3)

$f^{7}$ refers to the convolution operation with the kernel size of 7. Finally, the feature $F^{\prime }$ is scaled by $M_{s}$ as follows:

$$F^{\prime \prime }=M_{s}\left(F^{\prime }\right)⊗F^{\prime }\in R^{C\times d}$$ (4)

$F^{\prime \prime }$ is the final feature after CBAM block processing, and has adaptively selected the most important features and the most important part of each feature for classification.

#### 2.2.2 Multi-exit classifier module

The structure of the three classifiers is shown in Figure 1, and they are nearly identical, with each classifier containing two core blocks: SE block, transformer encoder block.

##### 2.2.2.1 SE block

Our SE block is proposed based on SENet (Hu et al., 2020) and different from their study, we extend the convolution and pooling operations to the 2D plane. Taking the EMG Classifier module as an example, given a feature map $F=\left[F_{EEG},F_{EOG},F_{EMG}\right]\in R^{L\times C\times d}$ , L is the type of input signal, the SE block can adaptively assign different weights to the three signals according to the importance of the EEG, EOG, and EMG signals to the classification task. The SE block is similar to the CBAM block. First, two convolution operations are applied to F such that $F^{\prime }=Conv2D_{2}\left(Conv2D_{1}\left(F\right)\right)$ and $F^{\prime }$ has the exact dimensions as the input feature map. Next, global average pooling is performed along the spatial dimension that shrinks $F^{\prime }\in R^{L\times C\times d}$ to $F_{avg}^{\prime }\in R^{L\times 1\times 1}$ , and two additional 2D convolutional layers replace the full connection layers in SENet to reconstruct $F_{avg}^{\prime }$ . The operations are formalized as follows:

$$M_{se}\left(F^{\prime }\right)=\sigma \left(Conv2D_{2}\left(Conv2D_{1}\left(AvgPool\left(F^{\prime }\right)\right)\right)\right)\in R^{L\times 1\times 1}$$ (5)

$M_{se}\left(F^{\prime }\right)$ refers to the feature map produced by $F^{\prime }$ after pooling and convolution operations. Then, the final output $F^{\prime \prime }$ of the SE block is:

$$F^{\prime \prime }=F⊕\left(M_{se}\left(F^{\prime }\right)⊗F^{\prime }\right)\in R^{L\times C\times d}$$ (6)

where ⊕ is the point-wise addition and ⊗ is the point-wise multiplication.

##### 2.2.2.2 Transformer encoder block

**FIGURE 4:**
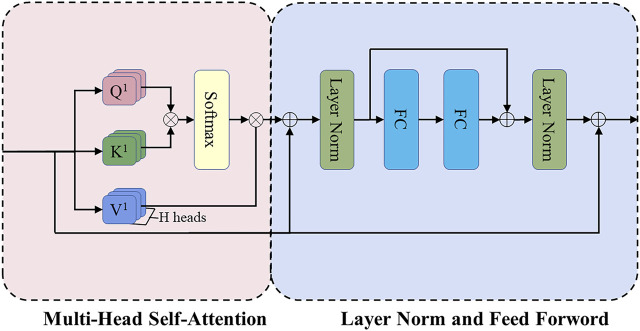
The structure of the Transformer encoder block. FC refers to the fully connected layer, and the position-wise feed-forward network (FF) consists of these two FC layers.

Figure 4 shows the structure of the Transformer encoder block. Multi-head self-attention has H headers, H different linear projections are applied to the input, and the result is mapped to parallel queries, keys, and values. Next, the dot-product is performed on $Q_{i}$ and $K_{i}$ to calculate a similarity score. A normalization operation is applied to stabilize the gradient. Then, the Softmax operation calculates the weight for $V_{i}$ , and another dot-product is applied. Finally, all the ${Attn}_{i}$ are concatenated together to produce the final output. The operations can be formulated as follows:

$$Q_{i}=XW_{i}^{Q},K_{i}=XW_{i}^{K},V_{i}=XW_{i}^{V},0<i\leq H$$ (7)

$$Attn_{i}=\sigma \left(\frac{Q_{i}\cdot K_{i}^{T}}{\sqrt{d}}\right)\cdot V_{i}$$ (8)

$$MHSA=\left[Attn_{1};Attn_{2};\ldots ;Attn_{H}\right]$$ (9)

where $X\in R^{LC\times d}$ is the input of the transformer encoder block, and LC refers to the product of signal type and feature. $W_{i}^{Q},W_{i}^{K},W_{i}^{V}\in R^{d\times \frac{d}{H}}$ are learnable weights of linear projections, d is the length of X . The operations of the layer normalization and position-wise feed-forward network are shown as follows:

$$IO_{1}=LayerNorm\left(MHSA⊕X\right)$$ (10)

$$IO_{2}=FC_{1}\left(FC_{0}\left(IO_{1}\right)\right)⊕IO_{1}$$ (11)

$$O=LayerNorm\left(IO_{2}\right)⊕X$$ (12)

Then O is fed into two linear layers for the final classification.

#### 2.2.3 Model training and inference

Dynamic neural networks (DNNs) aim to reduce the computational effort and increase the model generalization capability, and multi-exit is also one of the techniques of dynamic neural networks. The idea of multi-exit has been widely used in the fields of computer vision (CV) and natural language processing (NLP) (Liu et al., 2020; Wang et al., 2021), but to our knowledge, no multi-exit network has been specifically designed for the sleep stage classification task to reduce the computational cost of the network as of yet. In this section, we introduce the training and inference process of DynamicSleepNet.

##### 2.2.3.1 Single-exit training

Our model applies a three-step approach, namely “train twice, infer once”. The first training is consistent with the training process of most single-exit networks. This stage trains only three EFEMs and the EMG Classifier which is also the final classifier and the other classifiers are not enabled. EMG Classifier determines the class of the sample by using a combination of the three signals, so it has the highest classification accuracy.

##### 2.2.3.2 Multi-exit training

Unlike most single exit networks, the second training needs to freeze the parameters of the three EFEMs and the EMG Classifier, which means that this part of the network is not involved in this training. Only the EEG Classifier and the EOG Classifier are trained in this stage. The EEG Classifier uses only the EEG signal to classify samples, and the EOG Classifier uses only the EEG and EOG signals to classify samples, so their classification accuracy is lower than the EMG Classifier. To improve the accuracy of the two classifiers, we combined the self-distillation mechanism in the training process.

Knowledge distillation is a method of training a lightweight small model by using supervised information from a larger model with better performance (called teacher model) to enable the smaller model (called student model) to achieve better performance and accuracy (Goldberger et al., 2000; Gou et al., 2021). Unlike knowledge distillation, self-distillation does not require training with the aid of a large model, but uses the deeper network structures in its own model as the teacher model to train the shallower network structures in the model (Zhang et al., 2021). Inspired by the idea of self-distillation, we use the final classifier (EMG Classifier) of DynamicSleepNet as the teacher classifier and the two middle classifiers (EEG Classifier and EOG Classifier) as the student classifier. The training process in this stage is as follows.

The training samples are successively classified by the EEG Classifier, the EOG Classifier, and the EMG Classifier, and the students’ predictions $p_{s_{1}}$ and $p_{s_{2}}$ are compared with the teachers’ predictions $p_{t}$ respectively, with the differences measured by KL-Divergence in Eq. 13.

$$D_{KL}\left(p_{s},p_{t}\right)=\sum_{i=1}^{N}p_{s}\left(i\right)\cdot \log\frac{p_{s}\left(i\right)}{p_{t}\left(j\right)}$$ (13)

N refers to the number of classification categories, $p_{s}$ refers to the probability distribution of the output from student-classifier. The sum of the KL-Divergences of the two student classifiers is used as the total loss for self-distillation, as shown in Eq. 14.

$$Loss\left(p_{s_{1}},p_{s_{2}},p_{t}\right)=D_{KL}\left(p_{s_{1}},p_{t}\right)+D_{KL}\left(p_{s_{2}},p_{t}\right)$$ (14)

##### 2.2.3.3 Adaptive inference

With two training steps, DynamicSleepNet is ready to perform inference in an adaptive manner. At each classifier, we measure whether the inference for each sample is credible enough to be terminated early. Given an input sequence, the uncertainty of a student classifier’s output $p_{s}$ is computed with a normalized entropy in Eq. 15.

$$Uncertainty=\frac{\sum_{i=1}^{N}p_{s}\left(i\right)\log p_{s}\left(i\right)}{\log\frac{1}{N}}$$ (15)

For a classifier, the lower the uncertainty, the higher the probability that it is convinced the sample belongs to a certain class. When the uncertainty of a sample is lower than a threshold we set (we call it “Speed”), the sample will no longer be processed by the deeper network and will be directly classified as a class with the highest probability of classification, as shown in Figure 1.

Intuitively, with a higher Speed, fewer samples will be sent to deeper layers, and overall inference speed will be faster, and *vice versa*. Therefore, Speed can be used as a halt value for weighing the inference accuracy and efficiency.

## 3 Experiment

### 3.1 Baseline

Our model has been compared with six baseline models: AttnSleepNet, Epoch_CMT, MMASleepNet, DeepSleepNet-Lite, TinySleepNet, and SalientSleepNet. AttnSleepNet, Epoch_CMT, MMASleepNet and our model all adopt the model structure of CNN + Transformer with similar design concepts, and they are all single-sample input models. For these reasons we chose these three models to compare with our model: DeepSleepNet-Lite, TinySleepNet and SalientSleepNet are all models designed with lightweighting as a design concept, so we compared with the above three models in terms of both Parameters and Accuracy. Brief descriptions for these models are as follows:

**AttnSleepNet** (Eldele et al., 2021): AttnSleepNet uses CNN to extract features of EEG signals, followed by an adaptive feature recalibration module to distinguish the importance of features, and finally uses a multi-head self-attention mechanism for classification.

SleepPrintNet (Jia et al., 2020): A multimodal model with dedicated filters constructed for each of the three signals EEG, EOG, and EMG, emphasizing the differences in multimodal signal characteristics.

MMASleepNet (Zheng et al., 2022): Feature extraction is performed for the three signals of EEG, EOG, and EMG, and the attention mechanism is used to distinguish the importance of the three signals, and then using multi-headed self-attention for classification.

DeepSleepNet-Lite (Fiorillo et al., 2021): Using a lightweight feed-forward sleep scoring architecture and capturing samples with high uncertainty using Monte Carlo dropout sampling technique to improve the performance of the model.

TinySleepNet (Supratak and Guo, 2020): An efficient CNN + LSTM structure is used to classify single-channel EEG signals, which reduces the number of parameters and computational resources consumed by the model.

SalientSleepNet (Zheng et al., 2022): A U^2^-net based fully convolutional network to extract features from EEG, EOG signals. We use the revised SalientSleepNet as a comparison.

### 3.2 Evaluation metrics

Floating-point operations (FLOPs) is a measure of the computational complexity of a model, independent of the environment (CPU, GPU, TPU) in which the model is running, and it indicates the number of floating-point operations when a single process runs the model. With the same accuracy, the lower the FLOPs, the shorter the time taken for calculation and the more suitable for clinical use.

Parameters (Params) is a measure of how much memory a model takes up at runtime. If a device has less memory than the model needs to run, the model cannot run on the device. The lower the number of Params, the more suitable it is for deployment on small devices with low performance at the same accuracy.

The other three evaluation metrics are accuracy (ACC), macro-averaged F1-score (MF1), and Cohen’s Kappa (κ). Given True Positives ( ${TP}_{i}$ ), False Positives ( ${FP}_{i}$ ), True Negatives ( ${TN}_{i}$ ), and False Negatives ( $FN_{i}$ ) for the i-th class, ACC, MF1, and κ are defined as follows:

$$ACC=\frac{\sum_{t=1}^{T}TP_{t}}{N}$$ (16)

$$MF1=\frac{1}{T}\sum_{t=1}^{T}\frac{2\times \mathrm{Pr}e_{t}\times Rec_{t}}{\mathrm{Pr}e_{t}+Rec_{t}}$$ (17)

$$\kappa =\frac{ACC-p_{e}}{1-p_{e}}$$ (18)

where $p_{e}=\frac{\sum_{t=1}^{T}a_{t}\times b_{t}}{N\times N}$ , $\mathrm{Pr}e_{t}=\frac{TP_{t}}{TP_{t}\times FP_{t}}$ , $Rec_{t}=\frac{TP_{t}}{TP_{t}\times FN_{t}}$ . T refers to the number of classes, and N refers to the total number of samples. $a_{t}$ refers to the number of samples of class t, and $b_{t}$ refers to the number of samples predicted as the class t.

## 4 Results

### 4.1 Our model performance

**FIGURE 5:**
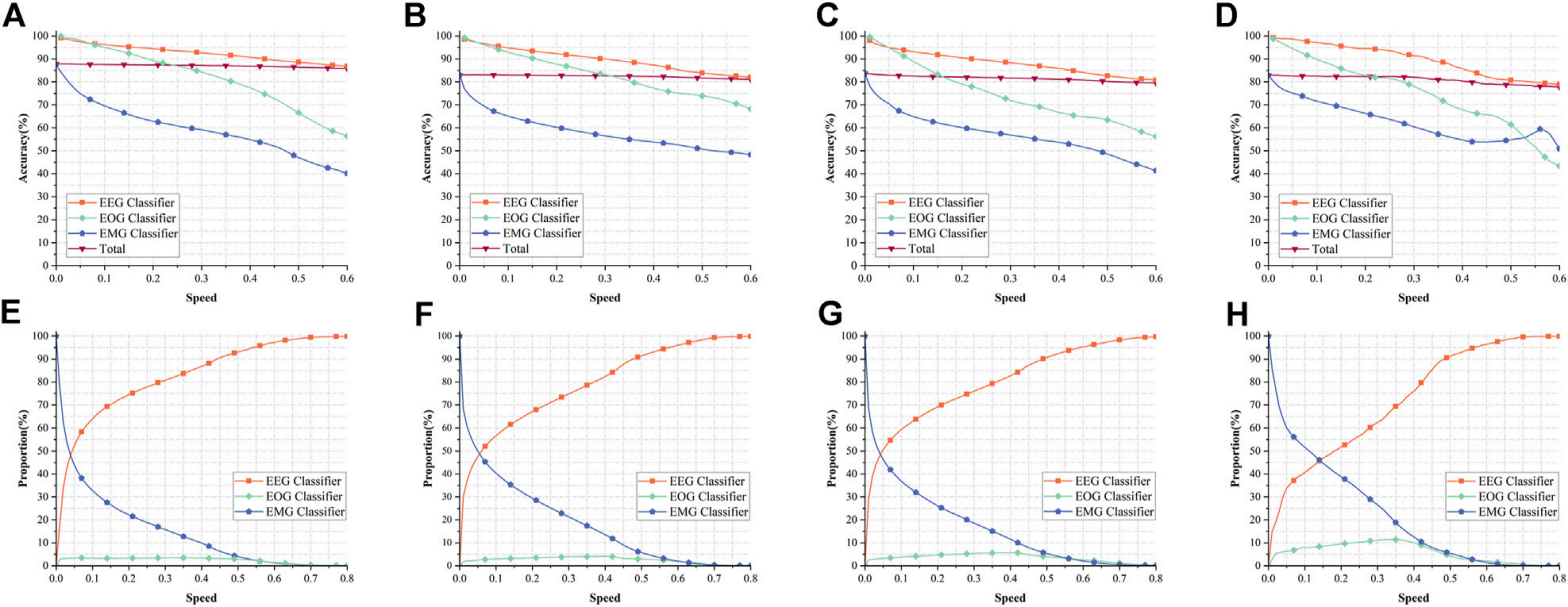
Performance of our model. **(A–D)** shows the variation curves of the accuracy of EEG classifier, the accuracy of EOG classifier, the accuracy of EMG classifier and the total accuracy in the four datasets of Sleep-EDF-20, Sleep-EDF-78, ISRUC-1, ISRUC-3, respectively. **(E–H)** correspond to **(A–D)** and show the percentage of samples output from the three classifiers in the four datasets.

Figures 5A–D show the variation curves of the total accuracy and the accuracy of EEG classifier, EOG classifier, and EMG classifier with the growth of Speed in the four datasets of Sleep-EDF-20, Sleep-EDF-78, ISRUC-1, and ISRUC-3 for our model, respectively. Overall, the accuracy of the EEG classifier is consistently higher than the other two classifiers, not because this classifier outperforms the other two classifiers, as we described in Section 2.2.3.3, when the uncertainty of a sample is lower than the Speed, that sample can be output earlier and does not need to go into a deeper network. Therefore, these samples with lower classification difficulty are outputted through the EEG classifier, resulting in the EEG classifier outperforming the other two classifiers. The EOG and EMG classifiers improve the performance of the classifier by training with the help of more kinds of information, but the samples that pass through these two classifiers are classified with higher difficulty, resulting in the poorer performance of the classifier. From Figure 7C, we can see that the performance of EMG and EOG classifiers is stronger than that of EEG classifier.

Figures 5E–H show the proportion of samples output from each exit in the four datasets Sleep-EDF-20, Sleep-EDF-78, ISRUC-1, ISRUC-3 to all samples. Comparing with Figures 5A–D, we can find that 71.11%, 55.27%, 50.50%, and 48.76% of the data in the four datasets can obtain more than 95% accuracy with very low computational resources consumption in the EEG classifier; With the uncertainty less than or equal to 0.05, 52.43%, 47.59%, 50.50%, and 33.71% of the data in the four datasets are also classified using the EEG classifier with 97.47%, 96.66%, 95.00%, and 98.58% accuracy at very low computational resource consumption, respectively. The above results show that the EEG classifier is sufficient to classify half of the data in Sleep-EDF-78, ISRUC-1, and ISRUC-3 with an accuracy of more than 95%, and nearly three-quarters of the data in Sleep-EDF-20 can be classified by the EEG classifier. In the Sleep-EDF-20, Sleep-EDF-78, and ISRUC-1 datasets, half of the data can be classified by the EEG classifier with higher accuracy, and more than one-third of the data in ISRUC-3 can be classified by the EEG classifier with an accuracy of 98.58%. These results prove our point in Section 1 that there are a large number of samples with low classification difficulty in the dataset that are sufficient for classification using only the EEG classifier, and adding more electrophysiological signals would only result in a significant waste of resources.

**FIGURE 6:**
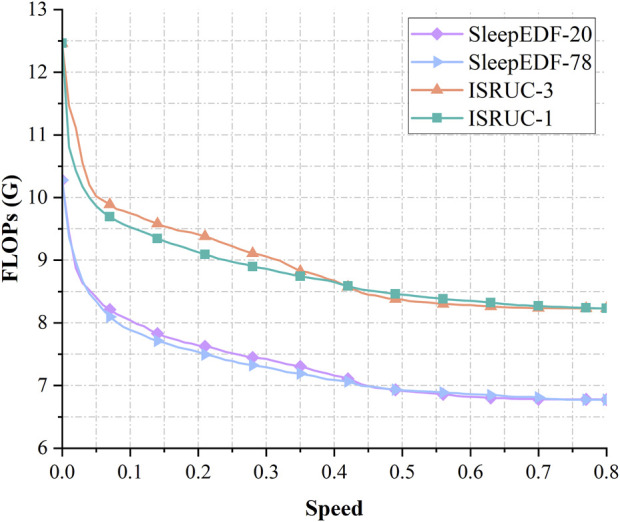
Variation curves of FLOPs with Speed in the four datasets of Sleep-EDF-20, Sleep-EDF-78, ISRUC-1, ISRUC-3.

We measured the FLOPs of the model in four datasets, as shown in Figure 6, under the condition of Speed = 0, all data are classified by the EMG classifier only, and the EEG and EOG classifiers do not perform any computation. The FLOPs of the model are about 10.28G in Sleep-EDF-20 and Sleep-EDF-78, and about 12.46G in ISRUC-1 and ISRUC-3. When the Speed increases to 0.1, a large number of samples with low classification difficulty are output early via the EEG and EOG classifiers without going through the whole network. The FLOPs of the model decreased by 21.76% and 23.26% in Sleep-EDF-20 and Sleep-EDF-78 to 8.04G and 7.88G, respectively, and by 23.58% and 21.77% in ISRUC-1 and ISRUC-3 to 9.53G and 9.75G, respectively, while the accuracy decreased by only 0.26%,0.093%,1.21%, and 0.37%, respectively. If the demand for accuracy is insensitive, the FLOPs of the model decrease by 32.74%,32.63%,32.19%,32.82% for 6.91G,6.92G,8.45G,8.37G in the four datasets with Speed = 0.5. Meanwhile, the accuracy decreases by 1.45%,1.35%,3.49%,4.07%. In Section 4.2 we will make a full comparison with other models.

### 4.2 Results comparison with baselines

Table 2 shows the results of our models compared with the baseline on three primary (ACC, MF1, κ) and five secondary (W, N1, N2, N3, and REM) metrics. Because not all baselines used the ISRUC-1 and ISRUC-3 datasets, our data are derived from other work (Zheng et al., 2022) complementing the baseline work. MMAsleepNet, SalientSleepNet, and SleepPtintNet are all multimodal models, therefore, on the whole, these three models perform better. Our model benefits from the use of attention-like mechanisms for channel, space, and signal type, so our model is slightly better than the other baselines in the Sleep-EDF-20 and Sleep-EDF-78 datasets with 87.86% and 83.03% accuracy, and has some advantages in other metrics as well. In the ISRUC-1 and ISRUC-3 datasets, our model has more obvious advantages, with the accuracy of 83.72% and 82.89%, and the other metrics are almost better than the baseline completely. These results show that our proposed model has better performance in the field of sleep stage classification.

**TABLE 2:** Comparison of DynamicSleepNet and baselines.

Dataset	Method	Per-class F1-score					Overall		
		W	N1	N2	N3	REM	ACC	MF1	κ
SleepEDF-20	AttnSleepNet	79.02	32.70	87.03	85.67	72.36	79.10	71.35	71.43
	MMASleepNet	92.20	54.75	**89.70**	**92.20**	86.41	87.30	82.65	82.63
	DeepSleepNet-Lite	87.10	44.40	87.90	88.20	82.40	84.00	78.00	78.00
	SalientSleepNet	90.79	49.86	89.03	84.77	**88.44**	86.28	80.58	81.02
	SleepPtintNet	88.77	47.99	86.72	86.21	80.26	83.08	77.99	76.67
	Ours (Speed = 0)	**93.41**	**57.15**	87.89	90.12	87.17	**87.86**	**83.15**	**83.47**
SleepEDF-78	AttnSleepNet	92.08	36.98	84.70	81.63	73.61	81.12	73.80	73.75
	MMASleepNet	**92.85**	49.05	84.94	81.26	79.75	82.67	77.60	76.12
	DeepSleepNet-Lite	91.50	46.00	82.90	79.20	76.40	80.30	75.20	73.00
	SalientSleepNet	92.28	**50.52**	84.37	71.17	**84.19**	82.61	76.51	75.92
	SleepPtintNet	79.12	40.12	58.22	68.80	73.67	81.64	63.99	56.02
	Ours (Speed = 0)	92.32	48.11	**85.77**	**82.19**	80.26	**83.03**	**77.73**	**76.44**
ISRUC-1	AttnSleepNet	84.19	43.80	71.52	81.93	61.12	71.65	68.53	63.70
	MMASleepNet	87.83	54.03	77.05	85.29	83.31	79.02	77.51	73.02
	DeepSleepNet-Lite	-	-	-	-	-	-	-	-
	SalientSleepNet	85.24	51.34	76.41	83.50	79.25	76.95	75.15	70.31
	SleepPtintNet	79.12	40.12	58.22	68.80	73.67	65.40	63.99	56.02
	Ours (Speed = 0)	**89.53**	**57.22**	**81.96**	**87.31**	**85.53**	**83.72**	**80.31**	**78.79**
ISRUC-3	AttnSleepNet	67.58	26.91	66.31	84.08	54.33	64.24	59.85	54.88
	MMASleepNet	88.87	59.57	**82.00**	87.00	**86.87**	81.92	80.64	76.79
	DeepSleepNet-Lite	-	-	-	-	-	-	-	-
	SalientSleepNet	78.37	50.64	77.33	87.99	75.47	76.11	73.96	69.39
	SleepPtintNet	85.15	52.53	74.95	87.28	74.84	76.88	74.95	70.29
	Ours (Speed = 0)	**89.86**	**62.39**	80.70	**90.68**	84.64	**82.89**	**81.65**	**79.08**

The best values on each dataset are highlighted in bold.

To validate the advantages of our model in terms of accuracy, speed and parameters, we use AttnSleepNet, SalientSleepNet, SleepPrintNet, and MMASleepNet for comparison with our model. Only AttnSleepNet is a single modal model, the other three are all multimodal models. To make the comparison fair, all data are measured in the Pytorch framework, and all parameters of the baseline model are kept the same as before. The number of samples entering into the model is uniformly set to 256. Due to the same sample structure of Sleep-EDF-20 and Sleep-EDF-78, and the same sample structure of ISRUC-1 and ISRUC-3, the baselines have the same results of FLOPs in Sleep-EDF-20 and Sleep-EDF-78, and the same results of FLOPs in ISRUC-1 and ISRUC-3. Our model is dynamic, and even if the datasets have the same structure, minor biases can occur due to the different depths when the samples exit the network. To avoid overly complicated table, we only show the FLOPs of Sleep-EDF-78 and ISRUC-1 because these two datasets have enough samples and are therefore more stable.

The comparison results are shown in Table 3. Our model is about 3 times faster than AttnSleepNet under the condition that the accuracy is higher or close to it, while the number of parameters is lower or approximate. It is 14.39 times faster and 23.81 times faster than SalientSleepNet under the condition that the accuracy is higher than it, while the number of parameters is close. While the accuracy is higher than SleepPrintNet, the speed is 3–5 times higher and the number of parameters is much lower. Under the condition that the accuracy is higher than MMASleepNet, the speed is 30%–90% higher than it while having a close number of parameters. These results are sufficient to show that our model is more efficient and more flexible to meet a wider range of needs in the field of sleep medicine.

**TABLE 3:** Comparison of Accuracy, FLOPs (speedup), and Params of DynamicSleepNet and baselines.

Method/Dataset	SleepEDF				ISRUC			
	Accuracy (20)	Accuracy (78)	FLOPs (Speedup)	Params	Accuracy (1)	Accuracy (3)	FLOPs	Params
**AttnSleepNet**	**79.10**	**81.12**	**20.32G**	**1.09M**	**71.65**	**64.24**	**26.26G**	**1.12M**
Ours (Speed = 0.00)	87.86 (+8.76)	83.03 (+1.91)	10.28G (1.98x)	1.29 M (+0.20)	83.72 (+12.07)	82.89 (+18.65)	12.40G (2.12x)	1.40 M (+0.28)
Ours (Speed = 0.10)	87.60 (+8.50)	82.93 (+1.81)	7.89G (2.56x)	1.29 M (+0.20)	82.50 (+10.85)	82.51 (+18.27)	9.53G (2.76x)	1.40 M (+0.28)
Ours (Speed = 0.60)	85.81 (+6.71)	81.09 (−0.03)	6.86G (2.96x)	0.97 M (−0.12)	79.54 (+7.89)	77.81 (+13.57)	8.35G (3.14x)	1.40 M (+0.28)
Ours (Speed = 0.73)	85.59 (+6.49)	80.45 (−0.67)	6.78G (3.00x)	0.97 M (−0.12)	79.15 (+7.50)	77.78 (+13.54)	8.26G (3.18x)	1.07 M (−0.05)
**SalientSleepNet**	**86.28**	**82.61**	**113.52G**	**1.18M**	**76.95**	**76.11**	**226.92G**	**1.18M**
Ours (Speed = 0.00)	87.86 (+1.58)	83.03 (+0.42)	10.28G (11.04x)	1.29 M (+0.11)	83.72 (+6.77)	82.89 (+6.78)	12.40G (18.30x)	1.40 M (+0.22)
Ours (Speed = 0.10)	87.60 (+0.32)	82.93 (+0.32)	7.89G (14.39x)	1.29 M (+0.11)	82.50 (+5.55)	82.51 (+6.40)	9.53G (23.81x)	1.40 M (+0.22)
Ours (Speed = 0.60)	85.81 (−0.47)	81.09 (−1.52)	6.86G (16.55x)	0.97 M (−0.21)	79.54 (+2.59)	77.81 (+1.70)	8.35G (27.18x)	1.40 M (+0.28)
Ours (Speed = 0.73)	85.59 (−0.69)	80.45 (−2.37)	6.78G (16.74x)	0.97 M (−0.21)	79.15 (+2.20)	77.78 (+1.67)	8.26G (27.47x)	1.07 M (−0.11)
**SleepPrintNet**	**83.08**	**81.64**	**21.54G**	**5.64M**	**65.40**	**76.88**	**48.31G**	**9.97M**
Ours (Speed = 0.00)	87.86 (+4.78)	83.03 (+1.39)	10.28G (2.10x)	1.29 M (−4.35)	83.72 (+18.32)	82.89 (+6.01)	12.40G (3.90x)	1.40 M (−8.57)
Ours (Speed = 0.10)	87.60 (+4.52)	82.93 (+1.29)	7.89G (2.73x)	1.29 M (−4.35)	82.50 (+17.10)	82.51 (+5.63)	9.53G (5.07x)	1.40 M (−8.57)
Ours (Speed = 0.60)	85.81 (+2.73)	81.09 (−0.55)	6.86G (3.14x)	0.97 M (−4.67)	79.54 (+14.14)	77.81 (+0.93)	8.35G (5.79x)	1.40 M (−8.57)
Ours (Speed = 0.73)	85.59 (+2.51)	80.45 (−1.19)	6.78G (3.18x)	0.97 M (−4.67)	79.15 (+13.75)	77.78 (+0.90)	8.26G (5.85x)	1.07 M (−8.90)
**MMASleepNet**	**87.30**	**82.67**	**10.22G**	**1.17M**	**79.02**	**81.92**	**18.23G**	**1.58M**
Ours (Speed = 0.00)	87.86 (+0.56)	83.03 (+0.36)	10.28G (0.99x)	1.29 M (+0.12)	83.72 (+4.70)	82.89 (+0.97)	12.40G (1.47x)	1.40 M (−0.18)
Ours (Speed = 0.10)	87.60 (+0.30)	82.93 (+0.26)	7.89G (1.30x)	1.29 M (+0.12)	82.50 (+3.48)	82.51 (+0.59)	9.53G (1.91x)	1.40 M (−0.18)
Ours (Speed = 0.60)	85.81 (−1.49)	81.09 (−1.58)	6.86G (1.49x)	0.97 M (−0.20)	79.54 (+0.52)	77.81 (−4.11)	8.35G (2.18x)	1.40 M (−0.18)
Ours (Speed = 0.73)	85.59 (−1.71)	80.45 (−2.22)	6.78G (1.51x)	0.97 M (−0.20)	79.15 (+0.13)	77.78 (−4.14)	8.26G (2.21x)	1.07 M (−0.51)

### 4.3 Ablation study

**FIGURE 7:**
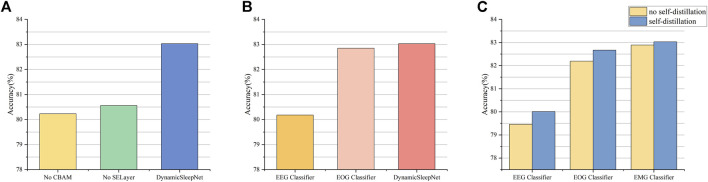
Results of ablation studies. **(A)** is the result of removing CBAM or SELayer compared with DynamicSleepNet. **(B)** is the result of classifying the Sleep-EDF-78 dataset using only the EEG classifier or using both EEG and EOG classifiers.**(C)** is the result of the comparison between not using self-distillation and using self-distillation.

CBAM and SELayer are two important blocks for extracting effective features, while adaptive multiple exits and self-distillation are two important mechanisms for achieving sample early exit, and to verify their effectiveness, we conduct ablation studies using the Sleep-EDF-78 dataset as an example. In order to be able to fully demonstrate the effectiveness of CBAM and SELayer, we will close all exits other than the final exit so that CBAM and SELayer can be maximally utilized. Figure 7A shows the results of removing CBAM and SELayer compared with DynamicSleepNet. The results show that the accuracy of the model drops dramatically after removing CBAM or SELayer, so CBAM and SELayer are very important for our model.

To research the effect of adaptive inference on our model, we conduct two experiments independently, one fixing all samples to be output only from the EEG classifier, and the other fixing all samples to be output both the EEG and EOG classifiers. The results of comparison with DynamicSleepNet are shown in Figure 7B. The results show that the adaptive inference mechanism enables a portion of the samples to enter the deeper layers of the network, thus improving the overall accuracy of the model.

Figure 7C shows the comparison results of EEG classifier and EOG classifier under the conditions without and with self-distillation, respectively, and the results show that self-distillation can improve the classification accuracy of early exits.

## 5 Conclusion and future work

In this study, we propose a multi-exit neural network with adaptive inference for sleep stage classification. By setting exits in different depths of the network and allowing samples to be output earlier, the classification speed of the model is greatly improved without much loss of accuracy. The model we designed allows simple samples with significant features to be output earlier, so we added CBAM blocks to the convolution process to make the effective features more prominent and the invalid features suppressed. In the first part of each classifier, we also use SELayer to enable the network to distinguish the importance of several electrophysiological signals for classification. In the ablation study, we demonstrated the effectiveness of these two modules. As far as the results are concerned, our proposed model outperforms all the bases in terms of accuracy. And under the condition that the accuracy is close, the computational speed of our model is several times faster than baselines and has a smaller number of parameters.

The results of this paper also point the way for our future work mainly on two points: 1) Improving the generalizability of our model. 2) Improving our model for a small number of samples that are difficult to be classified correctly. This is the key to improving the accuracy of the model.

## Data Availability

Publicly available datasets were analyzed in this study. This data can be found here: https://www.physionet.org/content/sleep-edfx/1.0.0/. The ISRUC datasets used in this paper can be found in https://sleeptight.isr.uc.pt/.
